# The effect of Norethisterone acetate on the uterus of albino rats: histological, histochemical and ultrastructure study

**DOI:** 10.1186/s12917-024-04219-0

**Published:** 2024-08-29

**Authors:** Mahmoud Abd-Elkareem, Sulaiman Mohammed Alnasser, Alotaibi Meshal, Raghda Ismail Abdullah, Ahmed U. Ali

**Affiliations:** 1 Department of Cell and Tissues, Faculty of Veterinary Medicine, Assiut University, Assiut, 71526 Egypt; 2 Department of Pharmacology and Toxicology, College of Pharmacy, Qassim University, Qassim, 51452 Saudi Arabia; 3 Pharmacy practice, College of pharmacy, University of Hafr Albatin, Hafr Albatin, Saudi Arabia; 4 Department of Anatomy, Histology and Embryology, Faculty of Veterinary Medicine, New Valley University, El Kharga, Egypt; 5 Department of Pharmaceutics, Faculty of Pharmacy, Merit University, Sohag, Egypt

**Keywords:** Uterus, Norethisterone acetate, Endometrium, Uterine glands, Rat, Apoptosis

## Abstract

**Background:**

Norethisterone acetate (NETA), also known as norethindrone acetate is a progestogens medication that is widely used in birth control pills, menopausal hormone therapy, and for the treatment of gynecological disorders as abnormal uterine bleeding and endometriosis. There is a lack of detailed histological information regarding the effects of NETA on the uterine structure. So, the present study focuses on the uterine histological, histochemical and ultrastructure changes following the exposure to NETA in the albino rats. To do this aim, fourteen adult female albino rats were used. They were randomly divided into two equally groups: Control group and NETA treated group. Albino rats of control group were administered daily food, water and orally distilled water only, while rats of NETA treated group were administered daily orally 20 µg of NETA dissolved in 2 ml distilled water, food, and water. The experiment was continued for three weeks.

**Results:**

The findings of the present work indicated that the use of NETA has negative effects on the endometrial epithelium (proliferation, autophagy and apoptosis), glands (necrotic, apoptotic or pseudosecretory glands) and stromal and myometrial reactions (granulocytes, connective tissue remodeling, apoptosis, myocytes hypertrophy).

**Conclusion:**

This work revealed that NETA has desynchronized progestogenic effect on the uterine tissues of the albino rat and thereby prevent implantation and pregnancy.

## Background

Progestogens (also known as progestins) are class of synthetic hormonal drugs that mimic progesterone’s endogenous hormone and widely used as contraceptives [1, 2]. Progestin-only contraceptive effectively and safely prevent pregnancy through desynchronization of the endometrial picture necessary for implantation [3, 4].

Norethisterone acetate (NETA), also known as norethindrone acetate is a potent contraceptive medication with a strong endometrial effect. It is widely used in birth control pills, menopausal hormone therapy, and for the treatment of gynecological disorders, such as abnormal uterine bleeding and endometriosis [5]. While all progestogens mimic natural progesterone, each of which has its ones characteristics based on the different pharmacokinetic and pharmacodynamic properties, including different binding affinities to estrogen, androgen, and glucocorticoid and mineralocorticoid receptors [6].

Norethisterone is a first-generation progestogen because it was the first synthetically produced orally active progestogen. Nortestosterone is the source of norethisterone. This indicates that, in contrast to natural progesterone and its other derivatives (dydrogesterone and medroxyprogesterone), it has some androgenic activity [7]. Norethisterone has an affinity for endometrial progesterone receptors similar to that of the natural ligand and so it can exerts secretory and proliferative endometrial changes [2, 7]. Progestational effects encompass the atresia of growing and antral follicles, the prevention of ovulation, and the gradual inactivation of endometrial tissues [8, 9]. Prolonged, high-dose treatment can lead to endometrial atrophy [9]. Previous studies have shown that the use of synthetic hormones may alter the effects of sex hormones on the uterine cycle and potentially resulting in adverse outcomes for the uterus [2, 10, 11]. By binding to progesterone receptors on target cells, norethisterone affects them and subsequently changes the target genes. Additionally, it results in a variety of endometrial changes such atrophy, irregular secretion, and restricted proliferation that makes the tissue unsuitable for implantation [3, 12, 13]. It was concluded that using NETA negatively impacts ovarian follicles, resulting in a decrease in their quantity and atresia in all phases [8].

Nevertheless, there exists a paucity of detailed histological information concerning the effects of NETA on uterine morphology. So, the aim of the present study is to give more information on the uterine histological, histochemical and ultrastructure changes following the exposure to NETA in the albino rats. This help to understand the pathophysiology of NETA on the uterus and its contraceptive mechanism.

## Materials and methods

### Source of the animals

Female albino rats were obtained from the animal house of the Faculty of Veterinary Medicine at Assiut University. The rats were fed a commercial fed and put in metal cages at room temperature with 12 h light: 12 h dark schedule during the period of the experiment. Water and food were allowed ad libitum. The experiment lasted for three weeks. The experimental protocol was approved by the Local Ethical Committee and by the Institutional Review Board of Molecular Biology Research and studies Institute, Assiut University (IORG0010947-22-2024) and was carried out in accordance with relevant guidelines and regulations. This research was done in compliance with the ARRIVE guidelines and regulations (https://arriveguidelines.org). All national and institutional guidelines for animal care and use have been followed throughout the study procedures.

### Drug

Norethisterone acetate was obtained from Chemical Industries Development (CID) Company, Giza, Egypt.

### Experimental design

A total of 14 non-pregnant female albino mature rats (average body weight of 150–180 g and an average age of 2–3 months) were randomly assigned into two groups, each consisting of seven animals.

#### Control group

In this group, mature rats were administered daily 2 ml distilled water and supplied with drinking water and a commercially pelleted diet for three weeks.

#### Norethisterone acetate (NETA) treated group

In this NETA treated group, each rat was orally (by stomach tube) administered daily 2 ml water solution containing 20 µg of NETA and supplied with drinking water and a commercially pelleted diet for three weeks. Being a potent drug and its dose is too low and couldn’t be weighed or adjusted for each animal, so NETA was diluted with inactive ingredients as lactose [14]. 4 mg of NETA was geometrically diluted with 996 mg of lactose so; 5 mg of this mixture (contains 20 µg of NETA) was dissolved in 2 ml distilled water.

### Histological preparation

At the end of the experiment, rats were euthanized by cervical dislocation and uteri were dissected and fixed in 10% neutral buffered formalin. The fixed materials were dehydrated in ascending grades of alcohol, cleared in methyl benzoate, and then embedded in paraplast. Paraffin sections of 5 μm in thickness were cut and stained with the following histological stains:

1. Haematoxylin and Eosin for general histological examination of the uterus [15]. Harris’s Haematoxylin is alum haematoxylin ripped with mercuric oxid. It is prepared from the following:

Haematoxylin 2.5 gm.

Absolute alcohol 25 ml.

Potassium alum 50gm.

Distilled water 500 ml.

Mercuric oxide 1.25gm.

Glacial acetic acid 20 ml.

Eosin prepared as 1% in distilled water.

#### Method of staining

1. Dewax sections in xylene.
2. Hydrate through graded alcohol to water.
3. Stain in haematoxylin for suitable time (30 s).
4. Wash in running tape water for 5 min or less until the sections become blue.
5. Differentiate in 1% acid alcohol (1% HCL in 70% alcohol 5–10 s (in case of over staining).
6. Wash well in tape water until the sections retain again blue for 5 min.
7. Stain in Eosin (30 s) and wash in distilled water.
8. Dehydrate through alcohol then clear in xylene and mount.

2. Periodic acid Schiff (PAS) technique for demonstration of glycoprotein and laminin [15].

### Method of staining

1. Dewax sections and bring to distilled water.
2. Treat with periodic acid for 2 min then wash well with several changes of distilled water.
3. Cover with Schiff’s reagent for 8 min.
4. Wash in running water 5–10 min.
5. Stain nuclei with Harris Haematoxylene.
6. Wash in tape water.
7. Rinse in absolute alcohol.
8. Clear in xylene and mount as desired.

3. Picro-Sirius red technique for detection of the distribution of collagen fibers in the uterus [16, 17]. Picro-Sirius red solution prepared as follow: 0.1% direct red 80 plus 0.1% fast green dissolved in saturated aqueous picric acid (1.2% picric acid in water). Deparaffinize/ dewax sections by Xylene then rehydrate in descending grads of ethanol and wash in distilled water. Immerse sections in picro Sirius red solution and stain for 60 min at room temperature. Rinse slides quickly in 2 changes of acetic acid solution. Rinse slide in distilled water. Dehydrate slides in 2 changes of absolute alcohol. Clear and mount slide using DPX. Collagen fibers appear red in color.
4. Orcien stain for detection of the distribution of elastic fibers in the uterus [18]. The protocol as follow: Deparaffinize and rehydrate in a descending alcohol series. Rinse in distilled water then add orcein solution for 30 min at room temperature. Then rinse in distilled water. Dehydrate in ethanol 95% and ethanol 100% for 1 min each (2X). Then clearing in xylene for 5 min (2X). Mount the xylene-wet slides with e.g. DPX and cover glass. Results: elastic fibers = red-brown.

### Immunohistochemical detection of apoptosis by caspase-3

For detection of apoptosis, we used rabbit polyclonal antibody against caspase-3 with dilution 1: 200 (Catalog No.: A11953), ABclonal, USA and poly Q stain 2 step detection system goat anti-mouse/rabbit HRP, peroxidase quench, DAB kit, quartett, Germany. The immunohistochemical protocol used was according to the company instructions and as our previous work [19, 20]. The fixed samples underwent ethanol dehydration, methyl benzoate clearing, and paraplast embedding. Xylene was used to dewaxe paraplast-embedded tissue Sect. (5 μm). After rehydrating the slides with 100%, 95%, 80%, and 70% ethanol, PBS (pH 7.4) was used to rinse them. By adding 3% hydrogen peroxide and then washing in PBS, endogenous peroxidase was inhibited. The slides were cooled to room temperature after being submerged in 10 mM sodium citrate buffer (pH 6.0) at 95–98 °C for 20 min in order to detect antigens. After that, sections were cleaned in PBS. Caspase-3 was immunoexpressed using rabbit polyclonal antibody against caspase-3 with dilution 1: 200 (Catalog No.: A11953), ABclonal, USA. Following that, sections were incubated at room temperature for 30 to 60 min with the primary antibodies. Then the slides were cleaned with PBS and following the manufacturer’s instructions of poly Q stain 2 step detection system goat anti-mouse/rabbit HRP, peroxidase quench, DAB kit, quartett, Germany. Harris hematoxylin was used as a counterstain on the sections for 30 s. Sections were then cleared in xylene, dehydrated with 95% and 100% ethanol, and mounted with DPX.

### Semithin sections and transmission electron microscopic preparations

Small pieces of uteri were fixed in 2.5% glutaraldehyde in phosphate buffer (PH 7.2) for 24 h. The fixed specimen were washed in 0.1 M phosphate buffer and then post-fixed in 1% osmium tetraoxide. The post-fixed specimen was dehydrated in ascending grads of alcohol and then embedded in araldite resin. Semi-thin Sect. (1 μm) in thickness were stained with 1% toluidine blue. Ultrathin sections were performed by a Reichert ultra- microtome and stained with uranyl acetate followed by lead nitrate. Ultrathin sections were examined and electron micrographs were taken using a Jeol Jem 1200 EX Transmission Electron Microscope at Electron microscope center of Assiut University [21].

Paraffin sections and semithin sections were examined by OLYMPUS BX51 microscope, and the photos were obtained by OLYMPUS DP72 camera adapted into the microscope.

### Histopathological scoring

1. **Damage scoring**: Damage score in percentages for each histomorphological changes in the NETA treated rats compared to control rats [16, 22] was done by this equation:

$$\text{Damage}\:\text{score}\:\text{percentage} =$$

$$\frac{\text{Number}\:\text{of}\:\text{animals}\:\text{showing}\:\text{the}\:\text{histomorphological}\:\text{change}}{\text{Total}\:\text{number}\:\text{of}\:\text{animals}}\:\times\:\text{100}$$

2. **Counting of caspase-3 positive cells per microscopic filed**: The number of caspase-3 positive surface and glandular epithelial cells, stromal cells and smooth muscle fibers of myometrium was counting per microscopic filed.

### Statistical analysis

The data were expressed as mean values ± stander error of means (S.E.) The data were subjected to statistical analysis using the independent-samples t-test SPSS 16. The significance value was set at (*P* < 0.05).

## Results

### Effect of NETA on general uterine histology

Our results revealed that the uteri in the rats of the control group showed the cyclic changes of the estrous cycle in rat. While the uteri in NETA treated group showed destructive or proliferative and secretory (pseudopregnancy) changes. Microscopically the uterine wall was formed of endometrium, myometrium and perimetrium. The Endometrium was consisted of lamina epithelialis and lamina propria. Lamina epithelialis was formed of simple stage dependent epithelium while lamina propria was composed of connective tissue contained endometrial (uterine) tubular glands. The myometrium is composed of both inner circular and outer longitudinal smooth muscle fibers, which are interspersed with small-sized arteries and veins. The perimetrium contained loose connective tissue covered with simple squamous epithelium.

**Fig. 1:**
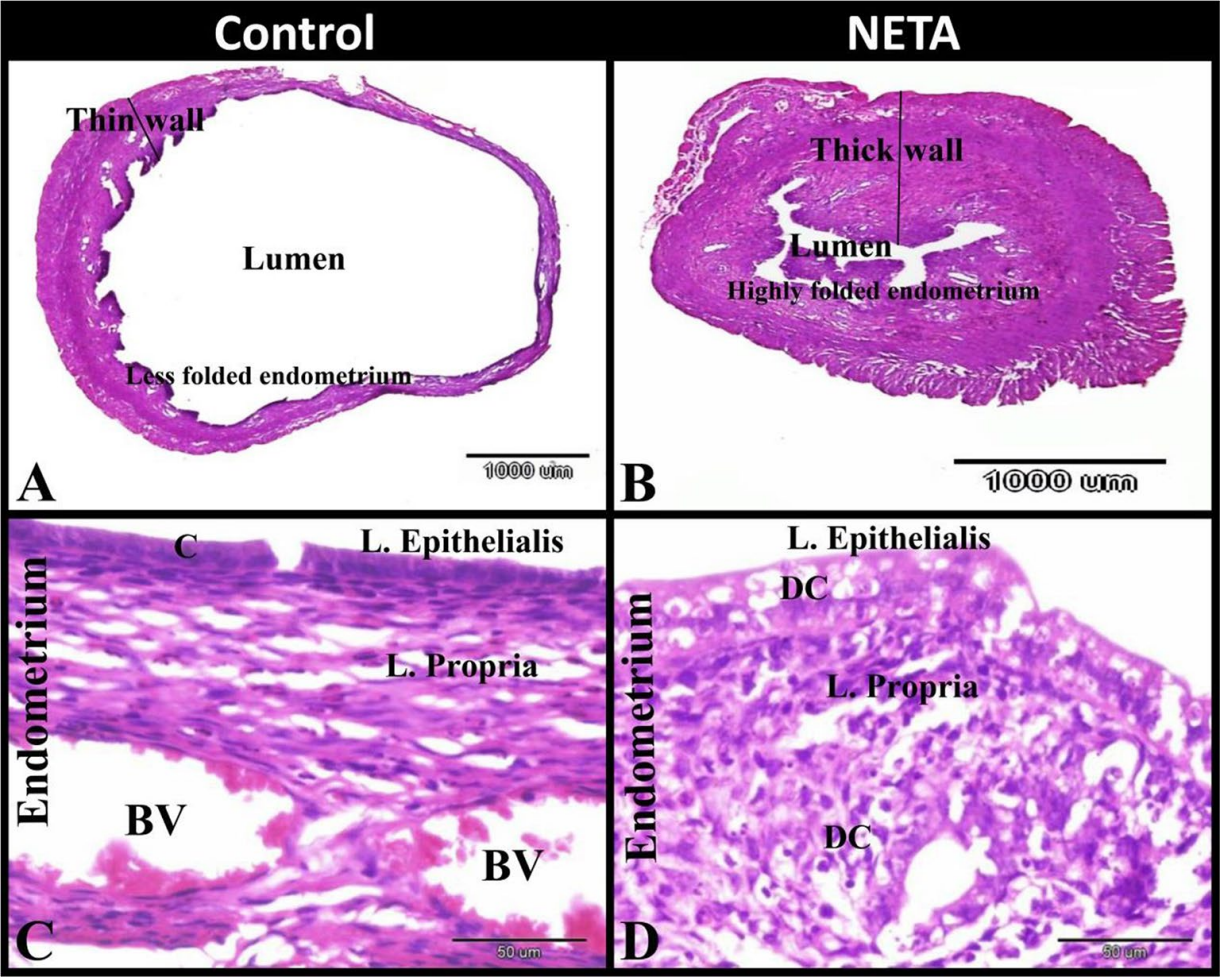
Photomicrograph of paraffin sections in rat uterus. **A**: Control group showing that the uterus had thin wall and wide lumen with less folded endometrium. **B**: NETA treated group showing that the uterus have thick wall and narrow lumen with highly folded endometrium. **C**: Control group showing that the endometrium was formed of lamina epithelialis of simple columnar epithelium (C) and lamina propria of dense connective tissue with fibroblasts and stroma cells and ectasic blood vessels (BV). NETA treated group showing that the endometrium was formed of lamina epithelialis of simple columnar or stratified epithelium with many dead autophagic cells (DC) and lamina propria of dense connective tissue with many dead stroma cells (DC). Original magnification; A & B: X12.5, scale bar = 1000 μm, **C & D**: X400, scale bar = 50 μm, Haematoxylin and Eosin stain

The control group showed that the uterus had thin wall and wide lumen with less folded endometrium. The endometrium was formed of lamina epithelialis of simple columnar epithelium and lamina propria of dense connective tissue with fibroblasts, stroma cells and ectasic blood vessels. While NETA treated group showed that the uterus had thick wall and narrow lumen with highly folded endometrium. The endometrium was formed of lamina epithelialis of simple columnar or stratified epithelium with many dead autophagic cells and lamina propria of dense connective tissue with many dead stroma cells (Fig. 1A-D).

**Fig. 2:**
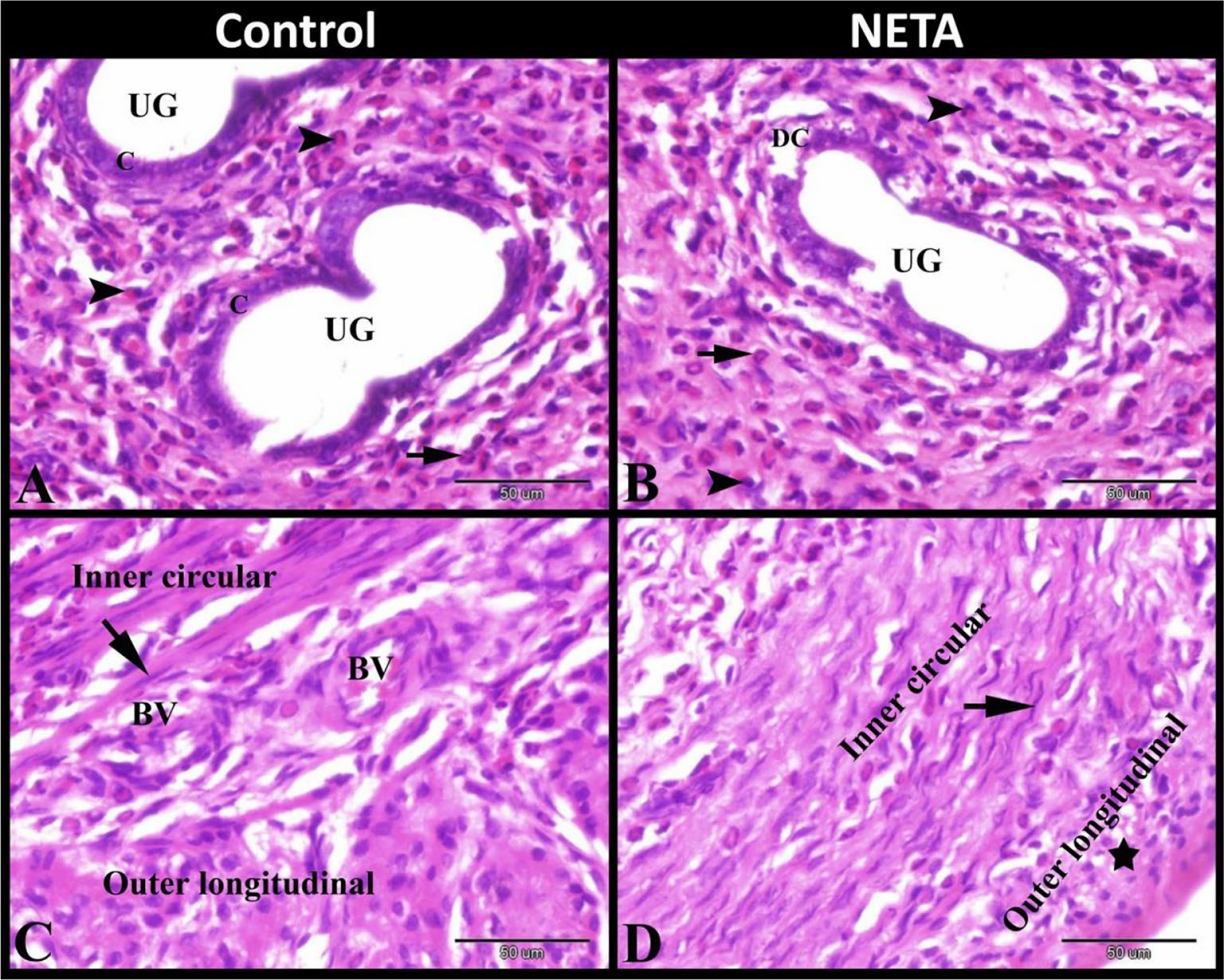
Photomicrograph of paraffin sections in rat uterus. **A**: Control group showing the endometrium contained uterine glands (UG) which formed of columnar epithelium (C) and contained polymorphnuclear leucocytes infiltration especially eosinophils (arrowheads) and neutrophils (arrow). **B**: NETA treated group showing the endometrium contained uterine glands (UG) with dead cells (DC) and contained polymorphnuclear leucocytes infiltration especially eosinophils (arrowheads) and neutrophils (arrow). **C**: Control group showing that the myometrium was formed of inner circular and outer longitudinal smooth muscle fibers with small sized blood vessels (BV) in-between. Note the spindle or rod shaped nuclei (arrow). **D**: NETA treated group showing that the myometrium was formed of inner circular smooth muscle fibers with some elongated irregular rod shaped nuclei (arrow) and outer longitudinal smooth muscle fibers with some vacuolar degenerated smooth muscle fibers. Original magnification; A-D: X400, scale bar = 50 μm, Haematoxylin and Eosin stain

In control group, the lamina propria of endometrium contained uterine glands which formed of columnar epithelium. There was Polymorphonuclear leukocytes (PMNs) infiltration especially eosinophils and neutrophils could be demonstrated in the lamina propria. While the myometrium was formed of inner circular (with spindle or rod shaped nuclei) and outer longitudinal smooth muscle fibers with small sized blood vessels in-between. In NETA treated group, the lamina propria of endometrium contained uterine glands with dead cells and also contained polymorphonuclear leukocytes infiltration especially eosinophils and neutrophils. While the myometrium was formed of inner circular smooth muscle fibers with some elongated irregular rod shaped nuclei and outer longitudinal smooth muscle fibers with some vacuolar degenerated smooth muscle fibers (Fig. 2A-D).

**Fig. 3:**
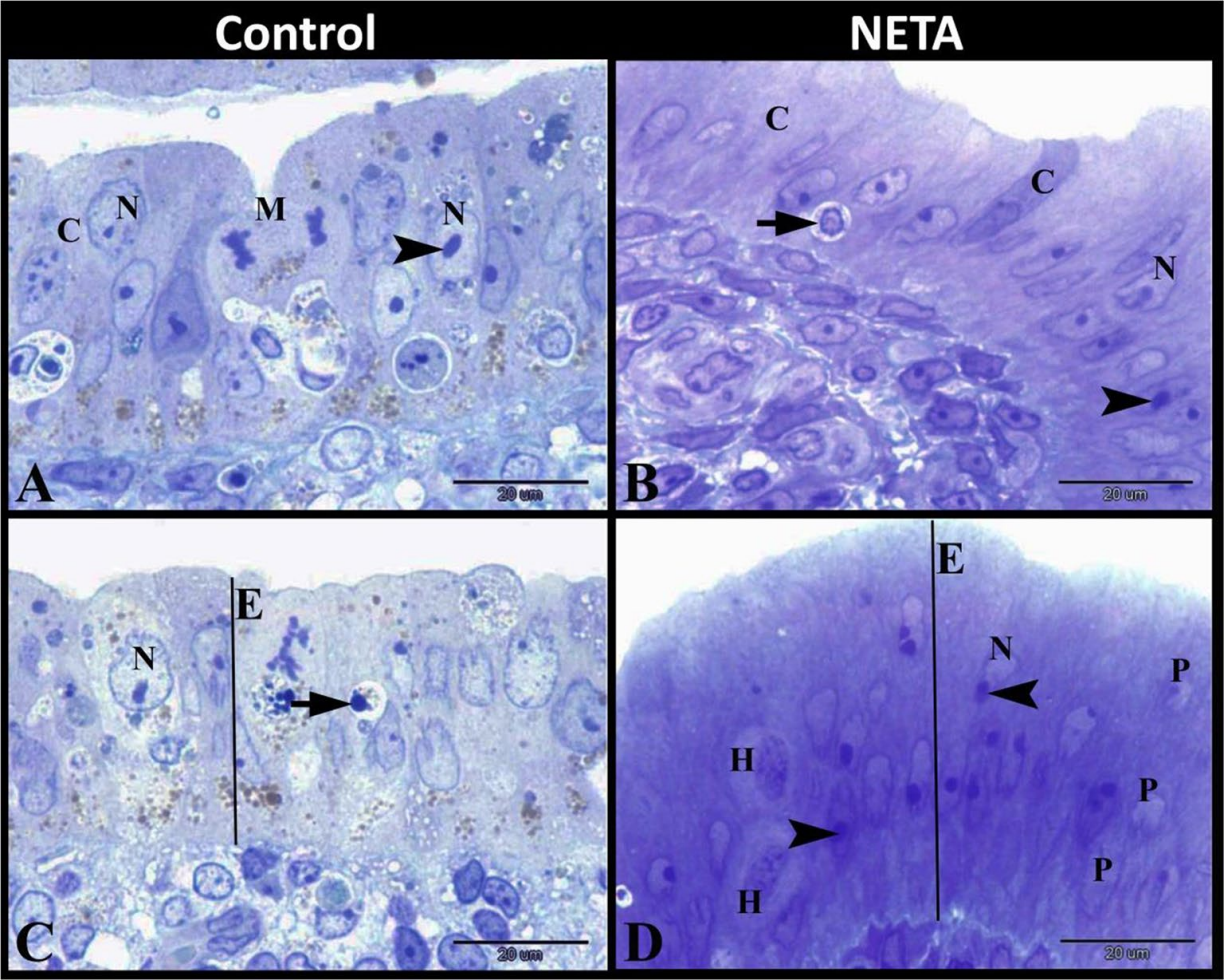
Photomicrograph of semi-thin sections in rat uterus. **A**: Control group showing the luminal epithelium which formed of pseudostratified columnar epithelium (C) with mitotic figures (M) and oval or rounded vesicular nuclei (N) with distinct central nucleoli (arrowhead). **B**: NETA treated group showing the luminal epithelium which formed of pseudostratified columnar epithelium (C) with no mitotic figures and elongated oval or irregular nuclei (N) with marginal nucleoli (arrowhead). Note the apoptotic cell (arrow). **C**: Control group showing the luminal epithelium (E) which had vesicular nuclei (N) and some apoptotic cells (arrow). **D**: NETA treated group showing increase the height of the luminal epithelium (E) by hypertrophy (H) and hyperplasia (P) to form epithelial tufting (E). Original magnification; A-D: X1000, scale bar = 20 μm, Toluidine blue stain

Our work revealed that the luminal epithelium in some animal of the control group was formed of pseudostratified columnar epithelium. This epithelium showed different forms of nuclei; oval or rounded vesicular nuclei with distinct central nucleoli while other revealed mitotic figures and some were apoptotic. Whereas the luminal epithelium in NETA treated group was formed of pseudostratified columnar epithelium with no mitotic figures and elongated oval or irregular nuclei with marginal nucleoli. Some apoptotic cells were also demonstrated in NETA treated group. In some areas the luminal epithelium showed an increase in the height by hypertrophy and hyperplasia of the epithelial cells to form epithelial tufting (Fig. 3A-D).

### Effect of NETA on mucopolysaccharides and laminin

**Fig. 4:**
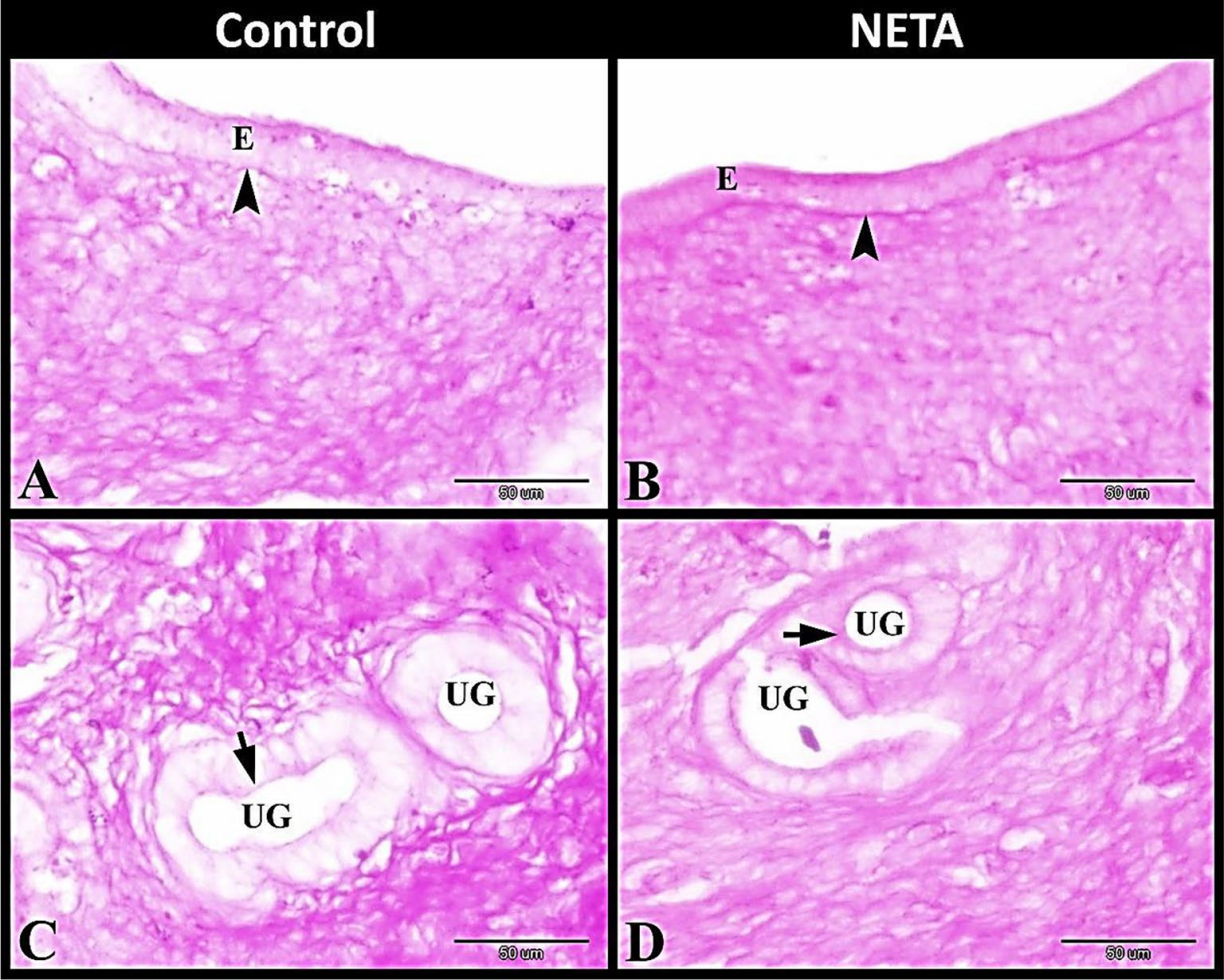
Photomicrograph of paraffin sections in rat uterus. **A**: Control group showing the luminal epithelium (E) had slight PAS positive materials and ill-defined PAS positive basement membrane (arrowhead). **B**: NETA treated group showing the luminal epithelium (E) had strong PAS positive materials and well-defined PAS positive basement membrane (arrowhead). **C**: Control group showing the uterine glands (UG) with slight PAS positive materials (arrow). **D**: NETA treated group showing the uterine glands (UG) with moderate PAS positive materials (arrow). Original magnification; **A-D**: X400, scale bar = 50 μm, Periodic Acid Schiff reagent (PAS) stain

Mucopolysaccharides and laminin investigation revealed that in the control group the luminal epithelium showed slight PAS positive materials and ill-defined PAS positive laminin containing basement membrane. Also the glandular epithelium showed slight PAS positive materials. In NETA treated group the luminal epithelium revealed strong PAS positive materials (Mucopolysaccharides) and well-defined PAS positive laminin containing basement membrane. While the glandular epithelium of NETA treated group had moderate PAS positive materials (Fig. 4A-D).

### Effect of NETA on uterine glands and collagen distribution

**Fig. 5:**
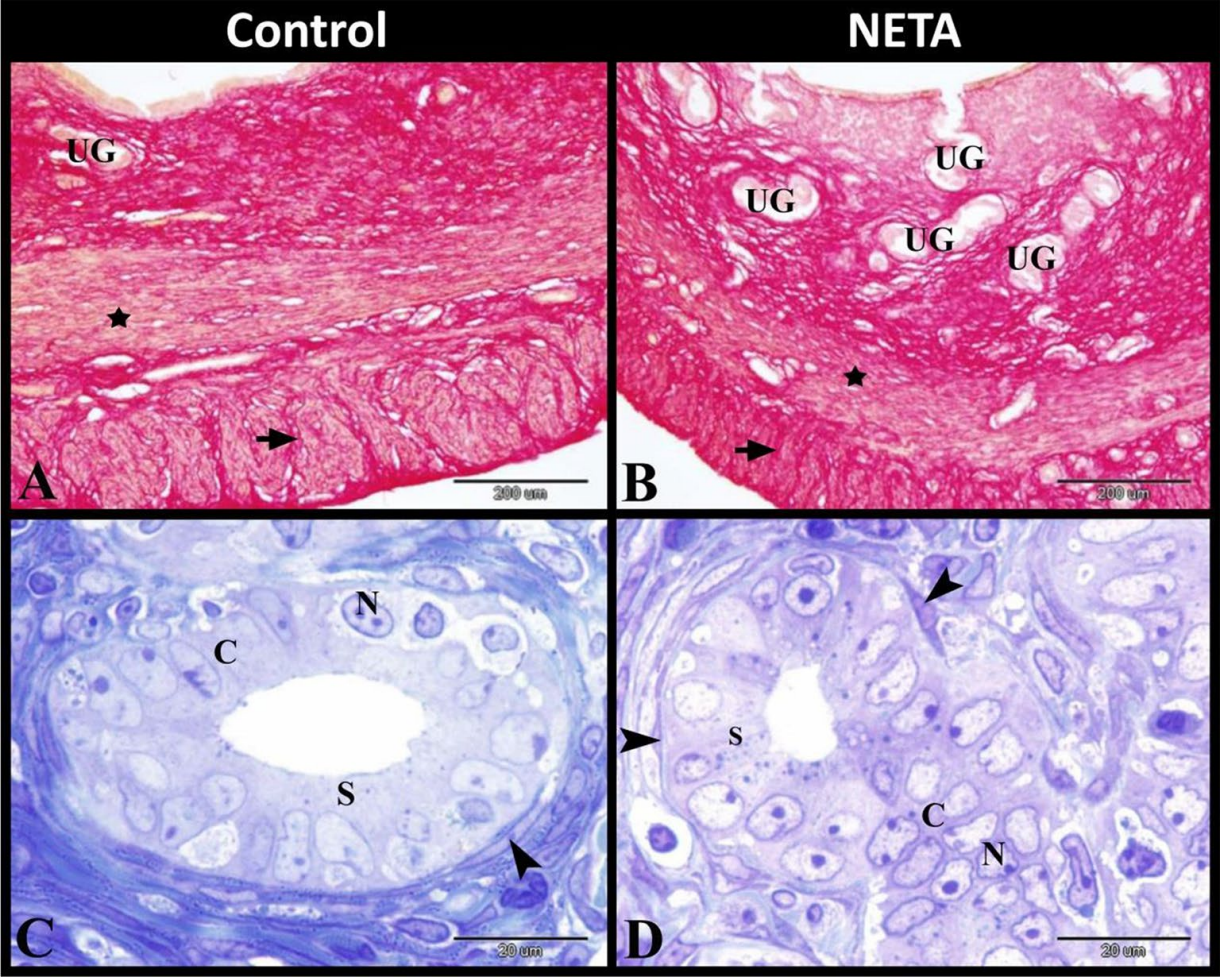
Photomicrograph of paraffin (**A & B**) and semi-thin (**C & D**) sections in rat uterus. **A**: Control group showing few uterine glands (UG) and normal collagen fibers distribution in inner circular (⋆) and outer longitudinal (arrow) smooth muscle layers of the myometrium. **B**: NETA treated group showing abundant uterine glands (UG) and increase collagen fibers distribution in inner circular (⋆) and outer longitudinal (arrow) smooth muscle layers of the myometrium. **C**: Control group showing the uterine glands formed of columnar epithelium (C) with oval vesicular nuclei (N) and few secretions (S). Note the myoepithelial cells (arrowhead) surrounding the uterine glands. **D**: NETA treated group showing the uterine glands formed of columnar proliferated epithelium (C) having irregular nuclei (N) with marginal nucleoli and abundant secretions (S). Note the myoepithelial cells (arrowheads) surrounding the uterine glands. Original magnification; A & B: X100, scale bar = 200 μm, Sirius red, C & D: X1000, scale bar = 20 μm, Toluidine blue stain

In this study we found that, in the control group there were few profiles of uterine (endometrial) glands and this was in contrast to many profiles in NETA treated group. We also demonstrated normal collagen fibers distribution in inner circular and outer longitudinal smooth muscle layers of the myometrium in control group. While in NETA treated group we observed an increase in the collagen fibers distribution in inner circular and outer longitudinal smooth muscle layers of the myometrium. The uterine gland in the control group was formed of columnar epithelium with oval vesicular nuclei and few secretions. Whereas the uterine gland in NETA treated group was formed of columnar proliferated epithelium which had irregular nuclei with marginal nucleoli and abundant secretions. In both groups the uterine glands were surrounded by myoepithelial cells (Fig. 5A-D).

### Effect of NETA on uterine muscles

**Fig. 6:**
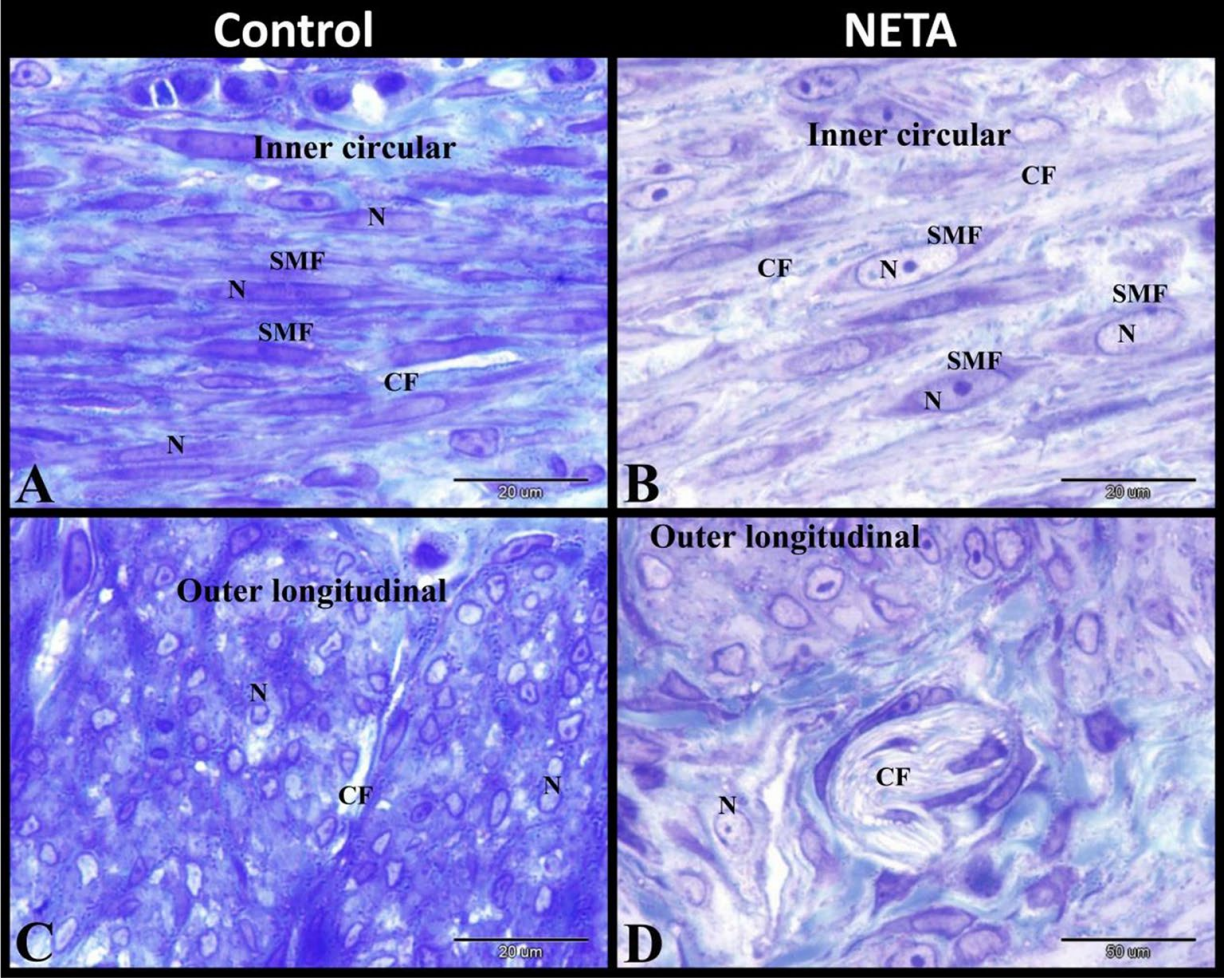
Photomicrograph of semi-thin sections in rat uterus. **A**: Control group showing normal spindle shaped or fusiform inner circular smooth muscle fibers (SMF) with spindle or rod shaped nuclei (N), and showing few collagen fibers (CF) in the myometrium. **B**: NETA treated group showing hypertrophied inner circular smooth muscle fibers (SMF) with large oval vesicular nuclei (N), and showing abundant collagen fibers (CF) in the myometrium. **C**: Control group showing normal rounded outer longitudinally smooth muscle fibers (of different sizes) with rounded nuclei (N), and showing few collagen fibers (CF) in the myometrium. **D**: NETA treated group showing hypertrophied outer longitudinally smooth muscle fibers with large rounded or irregular nuclei (N), and showing abundant collagen fibers (CF) in the myometrium. Original magnification; **A-D**: X1000, scale bar = 20 μm, Toluidine blue stain

Our findings revealed that there was different morphological appearance of smooth muscle fibers in the myometrium in both groups. In the control group the muscle fibers of the inner circular layer were normal spindle shaped or fusiform with spindle or rod shaped nuclei and this layer showed few collagen fibers (Fig. 6A). Whereas NETA treated group showed hypertrophied inner circular smooth muscle fibers with large oval vesicular nuclei and showed abundant collagen fibers in this layer of the myometrium (Fig. 6B). While the outer longitudinally smooth muscle fibers of the control group showed normal rounded structures of different sizes with rounded central nuclei and also this layer showed few collagen fibers (Fig. 6C). But NETA treated group showed hypertrophied outer longitudinally smooth muscle fibers with large rounded or irregular nuclei and abundant collagen fibers in this layer of the myometrium (Fig. 6D).

### Effect of NETA on uterine elastic fibers

**Fig. 7:**
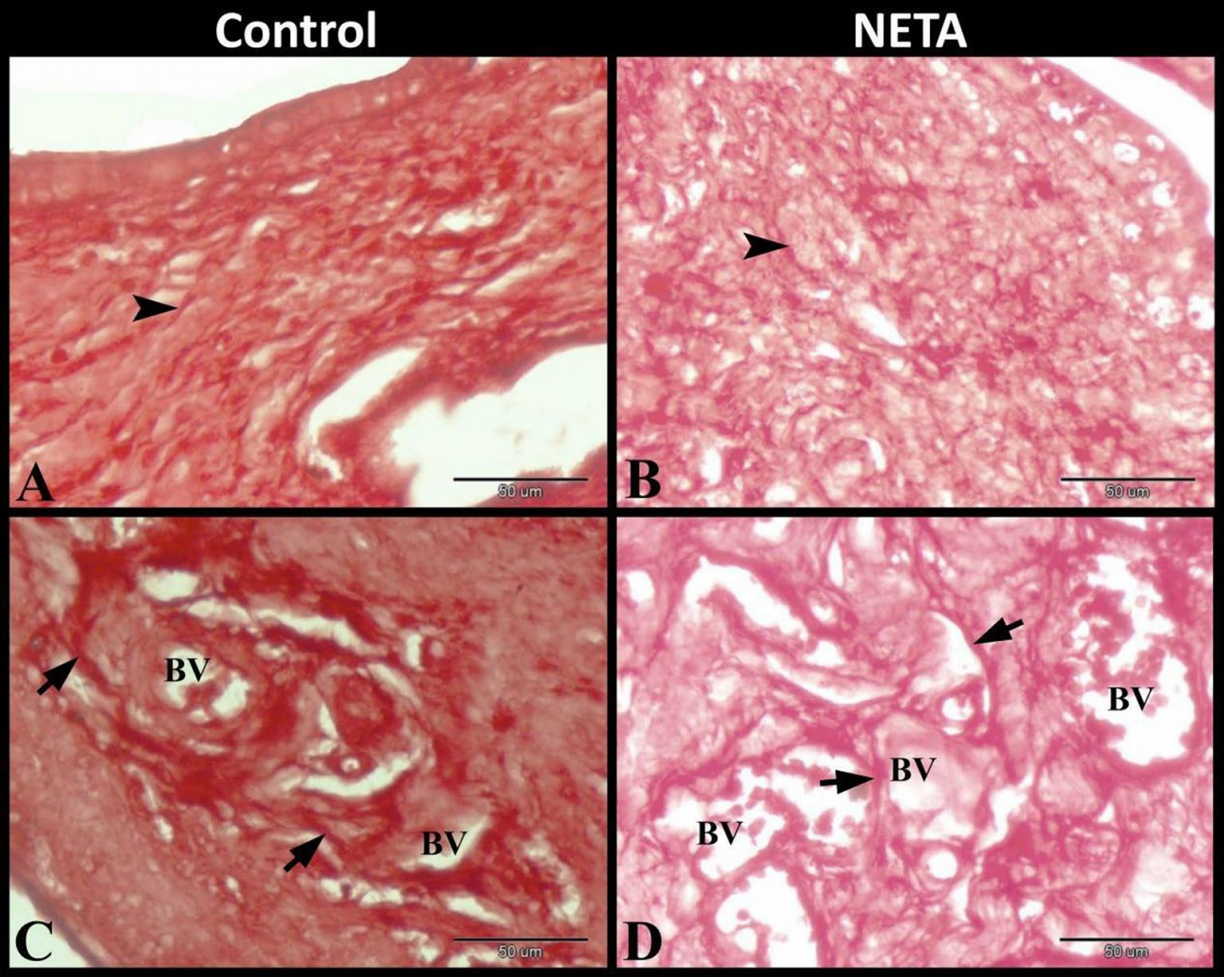
Photomicrograph of paraffin sections in rat uterus. **A**: Control group showing several elastic membranes (arrowhead) in the endometrium. **B**: NETA treated group showing scattered interconnected elastic membranes (arrowhead). **C**: Control group showing many elastic fibers (arrow) scattered in the myometrium especially in the wall of the small blood vessels (BV). **D**: NETA treated group showing increased amount of the elastic fibers (arrow) which scattered in the myometrium especially in the wall of the small blood vessels (BV). Original magnification; **A-D**: X400, scale bar = 50 μm, Orcein stain

Elastic fibers examination by orcein stain revealed that the control group showed several elastic membranes in the endometrium in addition to many elastic fibers were scattered in the myometrium especially in the wall of the small blood vessels. In NETA treated group the elastic fibers were present as scattered interconnected elastic membranes in the endometrium. While the myometrium revealed increased amount of the scattered elastic fibers which present especially in the wall of the small blood vessels (Fig. 7A-D).

### Effect of NETA on apoptosis

**Fig. 8:**
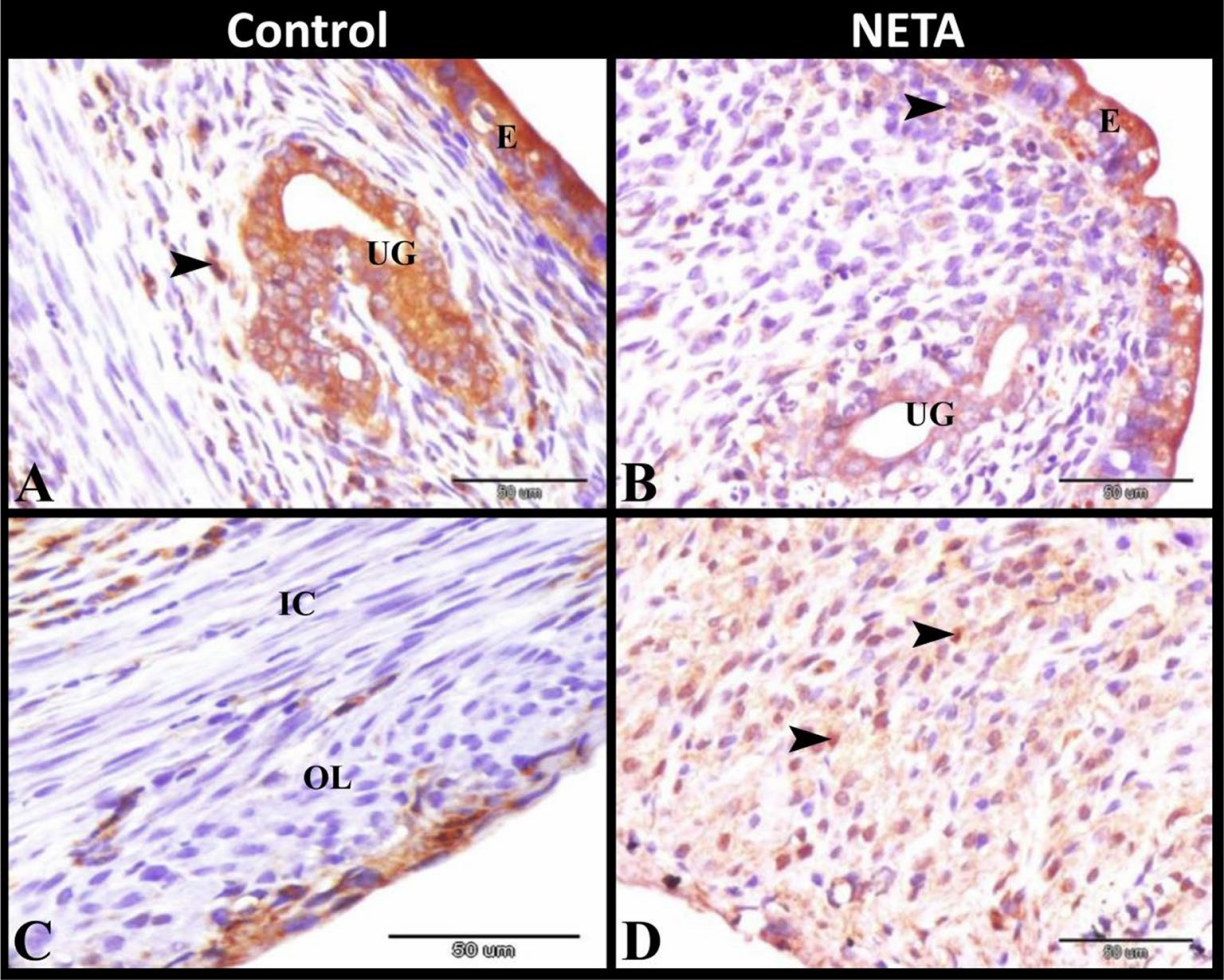
Photomicrograph of paraffin sections in rat uterus showing caspase-3 immunoexpression. **A**: Control group showing increased caspase-3 immunoexpression in luminal (E) and glandular epithelium (UG) and in some stroma cells (arrowhead). **B**: NETA treated group showing increased caspase-3 immunoexpression in luminal (E) and glandular epithelium (UG) and in some stroma cells (arrowhead). **C**: Control group showing caspase-3 negative immunostaining in the inner circular (IC) and outer longitudinal (OL) smooth muscles fibers of the myometrium. **D**: NETA treated group showing caspase-3 positive immunostaining in the outer longitudinal smooth muscles fibers (arrowheads) of the myometrium. Original magnification; **A-D**: X400, scale bar = 50 μm

**Fig. 9:**
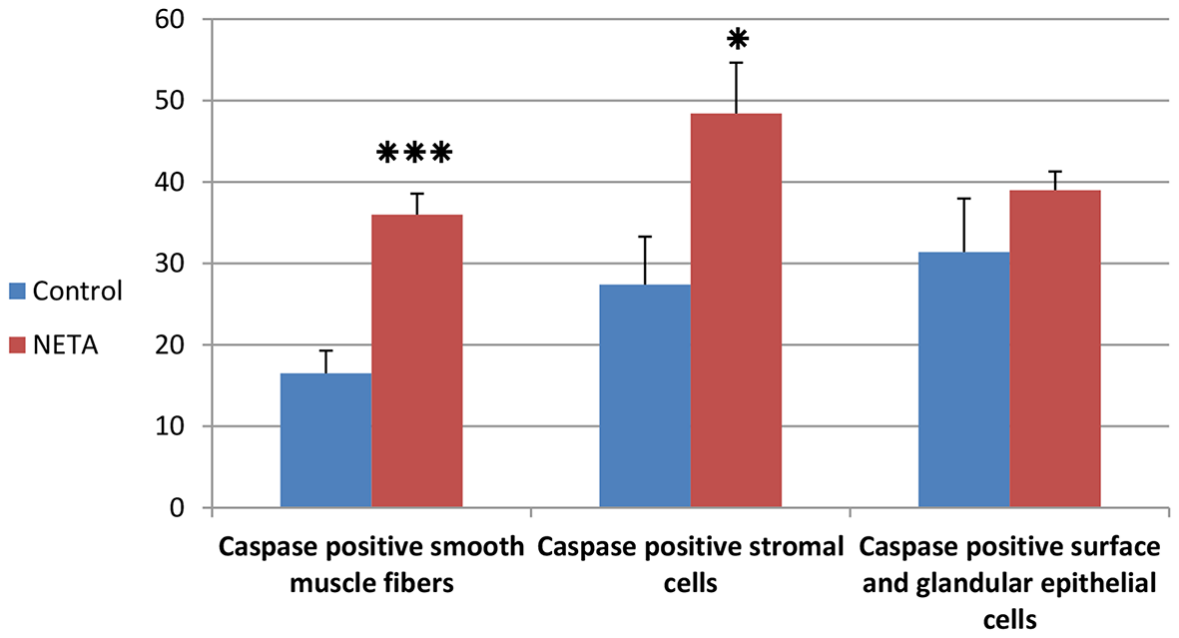
Showing the number of caspase-3 positive cells per microscopic filed. *** = statistically highly significance difference (*P* < 0.001) between NETA and control group. * = statistically significance difference (*P* < 0.05) between NETA and control group

Apoptosis detection by caspase-3 immunostaining revealed that there was an increase in caspase-3 immunoexpression in luminal and glandular epithelium and in some stroma cells in control (Fig. 8A) and NETA treated (Fig. 8B) groups. But there were caspase-3 negative immunostaining in the inner circular and outer longitudinal smooth muscles fibers of the myometrium in control group (Fig. 8C). While NETA treated group showed caspase-3 positive immunostaining in the smooth muscles fibers of the myometrium (Fig. 8D). Interestingly, we found that the number of caspase-3 positive smooth muscle fibers of the myometrium and stromal cells of the endometrium was significantly increased in NETA treated group compared to control. While the number of caspase-3 positive surface and glandular epithelial cells was non-significantly increased in NETA treated group compared to control Table 1 and (Fig. 9).

**Table 1:** Showing the number of caspase-3 positive cells per microscopic filed

Number of caspase-3 positive cells	Control	NETA
Surface and glandular epithelial cells	31.4 ^a^ ± 6.57	39 ^a^ ± 2.29
Stromal cells	27.4 ^a^ ± 5.89	48.42 ^b^ ± 6.24
Smooth muscle fibers of myometrium	16.5 ^a^ ± 2.79	36 ^b^ ± 2.57

Values (Means ± SE) with different superscripts (a and b) in the same row are significantly different (*P* < 0.05) between control and NETA groups

**Fig. 10:**
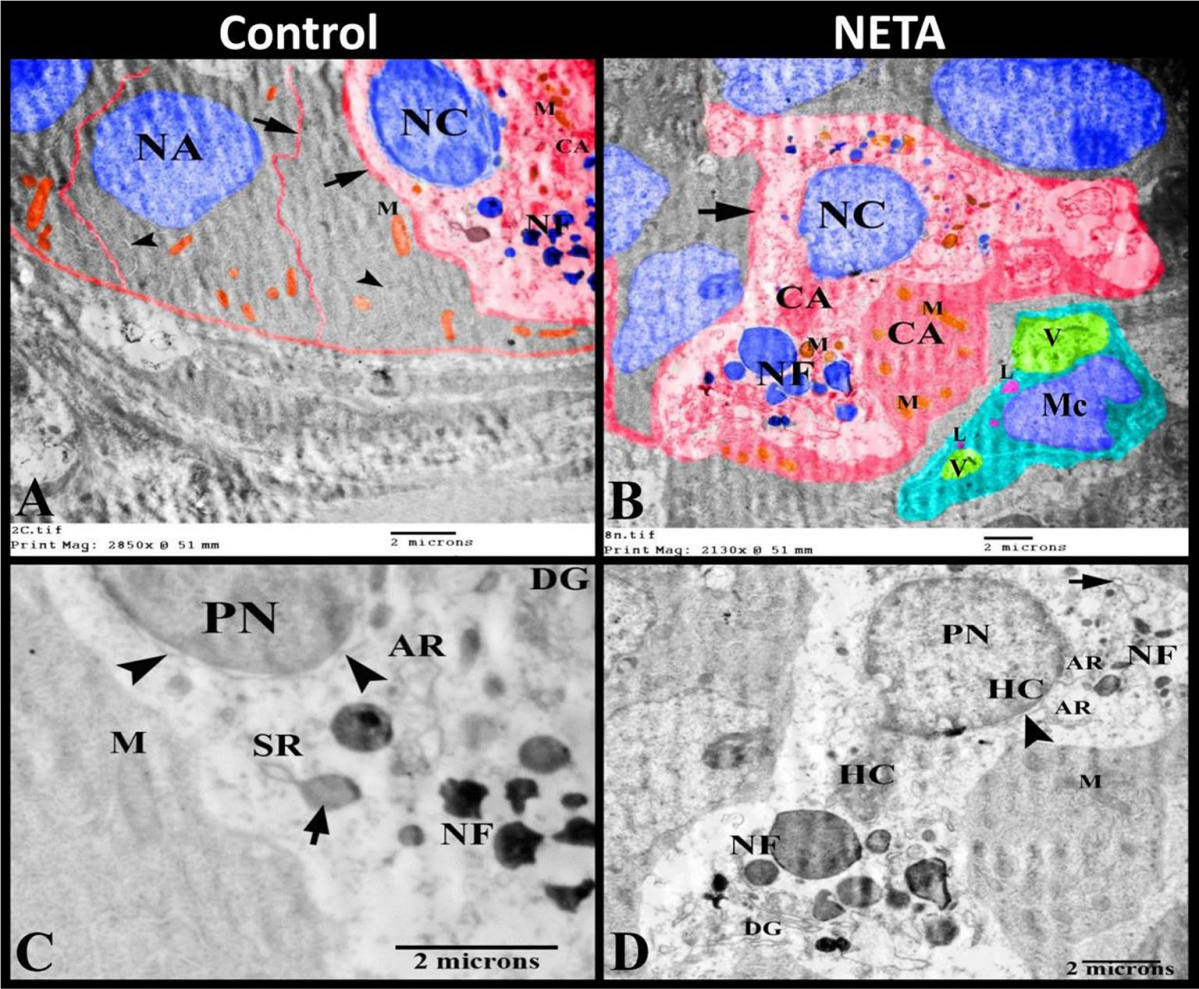
Photomicrograph of transmission electron microscopy in luminal and glandular epithelium of rat uterus. **A**: Control group showing signs of apoptosis; nuclear condensation (NC), nuclear (chromatin) aggregation (NA), nuclear fragmentation (NF), irregular corrugated plasma membranes (arrow), mitochondrial degradation (M), mirotubular disturbance (arrowheads) and cytoplasmic aggregation (CA). **B**: NETA treated group showing signs of apoptosis; nuclear condensation (NC), nuclear fragmentation (NF), irregular corrugated plasma membranes (arrow), mitochondrial degradation (M) and cytoplasmic aggregation (CA). Note the intraepithelial macrophage (Mc) with kidney shaped nucleus, primary lysosomes (L) and heterophagaic vacuoles (V). **C**: Higher magnification of Fig. A showing pyknotic nucleus, nuclear fragments (NF), irregular degraded outer nuclear membrane (arrowheads) with losing of its ribosomes, degraded Golgi apparatus (DG) and mitochondria (M), Apoptotic rough endoplasmic reticulum (AR), some of its cisternae were swollen (SR) by accumulations of proteins inside its lumens (arrow). **D**: Higher magnification of Fig. B showing pyknotic nucleus, increase the amount of heterochromatin (HC), nuclear fragments (NF), irregular degraded outer nuclear membrane (arrowhead) with losing of its ribosomes, degraded Golgi apparatus (DG) and mitochondria (M), Apoptotic rough endoplasmic reticulum (AR), some of its cisternae was swollen (arrow). Scale bar = 2 μm

Herein, by transmission electron microscopy the luminal and glandular epithelium of some control and most NETA treated rats showed the ultrastructural signs of apoptosis in the form of; nuclear condensation, nuclear (chromatin) aggregation, disappearance of the nucleolus, nuclear fragmentation (karyorrhexis), irregular corrugated plasma membranes, mitochondrial degradation, microtubular disturbance and cytoplasmic aggregation (Fig. 10A-B). Intaepithelial macrophages with its characteristic kidney shaped nucleus, primary lysosomes and heterophagic vacuoles could be observed in the NETA treated group (Fig. 10B). The pyknotic nucleus showed increase of the amount of heterochromatin, irregular degraded outer nuclear membrane with losing of its ribosomes. Golgi apparatus showed degraded and disassembles of its curved cisternae. While the mitochondria revealed disturbed outer and inner mitochondrial membranes and degraded cristae. In the apoptotic rough endoplasmic reticulum, some of its cisternae were swollen by accumulations of proteins inside its lumens (Fig. 10C-D).

The overall histomorphological alterations (lesion score) in the NETA treated rats compared to control rats were summarized in Table 2.

**Table 2:** Showing damage score in percentages for the histomorphological changes in the NETA treated rats compared to control rats

Histomorphological alteration	Control	NETA
**1- Endometrial epithelium**		
proliferation	25%	100%
autophagy or apoptosis	100%	50%
**2- Uterine glands**:		
proliferation	50%	100%
necrotic or apoptotic	100%	50%
pseudosecretory glands	0%	25%
**3- Stromal reactions**:		
polymorphnuclear leucocytes infiltration,	100%	100%
connective tissue proliferation,	50%	100%
stroma cell apoptosis	50%	50%
**4- Myometrial reactions**:		
polymorphnuclear leucocytes infiltration,	100%	100%
connective tissue proliferation,	50%	100%
apoptosis,	0%	25%
myocytes hypertrophy	0%	50%
**5- Thickening of the uterine wall**	50%	100%
**6- Widening of the uterine wall**	50%	0%

## Discussion

Our results revealed that the uteri in the rats of the control group showed the cyclic changes of the estrous cycle in rat and most of them were in estrous. While the uteri in NETA treated group showed destructive or proliferative and secretory (pseudopregnancy) changes. Herein, we found that the luminal and glandular epithelial cells of the endometrium expressed a cyclic process of proliferation, secretion and cellular death. It was found that epithelial death was included necrosis, apoptosis and autophagy. These processes were controlled by several ovarian hormones as progesterone and estrogens and played an indispensable role in the maintaining of the estrous cycle or embryo implantation and the maintaining of pregnancy.

In rat uterus, estrous was characterized by luminal dilatation and the appearance of cellular degeneration/necrosis in the glandular and endometrial epithelium. It also accompanied by a loss of mitotic activity and polymorph leucocytic infiltration; neutrophils and eosinophils [23, 24]. The morphological endometrial changes vary by the progestin contraceptive type, dosage and duration and whether or not estrogen is used. The prolonged use of combined oral contraceptive results in glandular and stromal atrophy and spiral arteriole underdevelopment. Whereas progestin-only implants result in atrophy with marked vascular changes like as underdevelopment of spiral arterioles and dilated, thin-walled vessels under the surface epithelium [25].

Observations of estrus cyclicity in progestin-only contraceptives treated rats showed a dose-dependent manner in the shift to a larger number of acyclic rats and prolonged diestrus phase [26]. Long-term administration of norethisterone leads to increase of epithelial height and/or epithelial percent in luminal and glandular epithelium of the uterus. It also increased the uterine myometrial percent with drop in stromal percent. Norethisterone suppressed the mitotic rate in all the regions of the female genital tract [27]. Some combined contraceptives which contained norethisterone and estradiol showed increased endometrial folding with dropped endometrial thickness. Luminal epithelium showed proliferation with pseudostratification, necrotic changes, hyperplasia (epithelial tufting). Furthermore, there was a reported incresed in the size of the gland and in the stromal hypercellularity. The presence of polymorphonuclear cellular infiltration in both the endometrium and myometrium, together with vascular congestion and increased thickness of the myometrium, were also observed [28]. Progesterone and other synthetic progestin exerts its effect on the uterus through binding to progesterone receptors [28].

Endometrial (uterine) glands are developing postnatally and present in all mammalian uteri. They produce and transport the uterine secretions which including leukemia inhibitory factor (LIF) and calcitonin (CALCA), that are important for implantation. Therefore, the absence or atrophy of uterine glands results in infertility in many species [29]. Several genes, including as forkhead box A2, beta-catenin, and various members of the *Wnt* and *Hox* gene families, play a crucial role in the development of uterine glands. Progestins prevent uterine epithelial proliferation, and so inhibit endometrial gland adenogenesis leading to infertility [30] and this may be due to its effect in the aforementioned genes.

The endometrial tissue is a crucial recipient of sex hormones and has the ability to promptly and flexibly adjust its histological properties. Oral contraceptives exert a significant progestational influence on the endometrium, resulting in the inhibition of glandular proliferation (inactive, atrophic), the development of pseudosecretion, and the occurrence of stromal edema. This is then followed by the transformation of the stroma into decidualized tissue, characterized by the presence of granulocytes and thin sinusoidal blood vessels. Additionally, they can lead to a decidual reaction in the absence of spiral arterioles [31, 32].

The contraceptive effects of synthetic progestins were mediated through three main mechanisms: anti-gonadotrophic action leading to the prevention of ovulation, alter the cervical mucus properties leading to inhibition of sperm penetration and desynchronization of the uterine picture necessary for implantation [3, 27, 31]. Most of progestogen only contraceptive showed one or more of three morphologic patterns of response which may be coexistent and overlapping depending on: dose and duration of progestin as well as endogenous estrogen levels. These morphologic patterns are decidual (pregnancy-like) pattern, secretory (luteal phase-like) pattern and inactive pattern (atrophic) [3, 31].

The regulation of uterine cell death and proliferation patterns is a crucial aspect of the sexual cycle and pregnancy-related uterine modifications, characterized by a well-organized and cell-specific changes [33]. Mitotic rates and apoptosis in rat uterine luminal and glandular epithelial cells were dynamic processes with mitotic rates increased in diestrous and the apoptotic index peaks at estrus [34]. Apoptosis plays a crucial role in the uterine environment by ensuring the maintenance of cell number balance throughout the estrous cycle and facilitating tissue remodeling during the process of implantation [35].

Estrogen and progesterone hormones are the main regulators of not only the proliferation and differentiation of luminal and glandular epithelial cells during the estrous cycle, but also their death by apoptosis [35].

There was a strong positive relation between autophagy and apoptosis in the endometrial epithelial cells. As in the late endometrial cell cycle, the accumulation of autophagosomes as a result of inhibition of autophagosome degradation by fusion with lysosomes lead to induction of apoptosis [36]. Many accumulating evidence indicated that endometrial autophagy which occurred under the regulation of ovarian hormones, can result in the leucocytic infiltration. These immune cells plays an indispensable role in the endometrium remodeling [37]. Autophagy is a highly conserved biological process in eukaryotic cells for the disposal of dysfunctional components, and misfolded proteins, aging organelles, and other damaged cell components to maintain the homeostasis of the cells. It was initiated under the conditions of hypoxia, starvation, lack of nutrition, or extreme pH values [38]. Autophagy has been identified as a significant contributor to several physiological and pathological processes in the endometrium, a highly efficient self-regenerating tissue within the human body. It also act as an instrumental player in the implantation and placentation and other uterine function [39].

Our study showed increased Caspase-3 immunoexpression in luminal and glandular epithelium, stroma cells and myocytes of NETA treated rats. In hamsters and mice there is a direct correlation between apoptosis and caspase-3 expression, proposing that uterine cell death mainly involves the caspase pathway [33]. Caspase-3 is belonging to family of Cysteine-ASPartic proteASES (cysteine proteases) which have the ability to mediate the cleavage of specific target proteins. Caspase-3 is a death protease that is often triggered and plays a key role in apoptosis. It is catalyzing the specific cleavage of many key cellular proteins [40, 41].

It was postulated that apoptotic cell death was an important regulatory factor for uterine remodeling prior to and during implantation in rats [42]. The hallmark of apoptosis is including: cell shrinkage, cytoplasmic and nuclear condensation (pyknosis), nuclear fragmentation (karyorrhexis), then the cell disaggregates into a number of membrane-bound apoptotic bodies, which are engulfed via phagocytosis by macrophages. Autophagy is manifested by the accumulation of cytoplasmic vacuoles and membranes [43–45]. Our findings by transmission electron microscopy revealed that the luminal and glandular epithelium of some control and most NETA treated rats showed the ultrastructural signs of apoptosis. These ultrastructure manifestation of apoptosis were; nuclear condensation, nuclear (chromatin) aggregation, nuclear fragmentation (karyorrhexis), irregular corrugated plasma membranes, mitochondrial degradation, microtubular disturbance and cytoplasmic aggregation. The pyknotic nucleus showed increase of the amount of heterochromatin, disappearance of the nucleolus, irregular degraded outer nuclear membrane with losing of its ribosomes.

Golgi apparatus showed degraded and disassembles of its curved cisternae. While the mitochondria revealed disturbed outer and inner mitochondrial membranes and degraded cristae. In the apoptotic rough endoplasmic reticulum, some of its cisternae were swollen by accumulations of proteins inside its lumens [43, 46, 47]. Morphological transformation in the mitochondria initiates apoptosis through the release of proteins such as cytochrome c from the intermembranous and intracristal spaces [48].

Intaepithelial macrophages with its characteristic kidney shaped nucleus, primary lysosomes and heterophagic vacuoles could be observed in the NETA treated group in the present study. Macrophages and other immune cells are important for getting rid of dead cells and cell debris during estrous cycle or early pregnancy. Clearance of the apoptotic cells and cell debris is a crucial event during uterine remodeling, as it maintain tissue homeostasis and protect the fetus [49].

Macrophages are professional phagocytes and are present around the rat uterine lumen and within the metrial gland during mid-pregnancy and postpartum. They might be involved in the clearance of apoptotic cells by efferocytosis [50]. Efferocytosis is a mechanically process for the effective clearance of apoptotic cells and cellular debris by phagocytes. This process involves the localization, binding, internalization, and degradation of apoptotic cells [51]. In macrophage, we found that the engulfed apoptotic cells and cellular debris were enclosed within a membrane-bound vacuole called a phagosome (hetero-phagosomes). Hetero-phagosomes were fused with the primary lysosome which contained hydrolytic enzymes. Then the macrophage digested and hydrolyzed the ingested material with hydrolytic enzymes.

Our results showed vacuolar degeneration and apoptosis in uterine myocytes of NETA treated rats. It was postulated that uterine caspase-3 maintained the uterine quiescence by acts as an anticontractile agent through fragmentation of uterine myocyte contractile proteins. In mouse this anticontractile action of caspase-3 was regulated by progesterone [52]. Fibroblast and smooth muscle cell proliferation was highest early in pregnancy and gradually dropped. Apoptosis, on the other hand, increased gradually as pregnancy went on. [53]. Contraceptive pills administration specially the combined one caused marked changes in the form of hyperplasia in uterine epithelial cells and hypertrophy of smooth muscles fibers in muscular layers. Also, lead to significant increase in collagenous and elastic fibers content in myometrium. There was also a significant increase in the PAS reaction in the lumen of the endometrial glands in uterus indicated secretory activity [54, 55]. Norethindrone acetate, suppress ovulation and endometrial growth and in some cases lead to secretory maturation [3, 27].

Our observations demonstrated that there was different collagen, elastin and laminin extracellular formation in control and NETA treated rats. We proposed that these differences in the synthesis of these extracellular proteins play indispensable role in extracellular matrix remodeling which is necessary for prevention or maintenance of pregnancy. During development of the pregnant rat uterus there was a significant increase in the elastin fibers in the extracellular matrix of the myometrium which follow random configurations. These elastic fibers may be important in the normal remodeling process of uterine connective tissue and extracellular matrix [56]. In the rat uterine wall, these elastic fibers were arranged parallel to the plane of the uterine surface in the form of highly branching elastic membranous sheets and were responsible for the flexibility and pliancy of this organ [57]. In mice, remodeling of the uterine extracellular matrix was characterized by synthesis, degradation and alteration of collagen and elastic fibers. Elastic fibers were formed of matrix glycoprotein; Fibrillin-1 which promotes cell adhesion through its integrin-binding domain. Changes in the expression of fibrillin-1 during the peri-implantation period suggested that the elastic fibers plays a role in preparing the endometrium for embryo implantation [58]. It was also found that collagen play an important role at the maternal-fetal interface in human pregnancy [59]. Some combined contraceptives caused reduction in the amount of uterine collagen fibers [28] and other like norethisterone acetate increase them as in this study.

Laminins are glycoproteins of the extracellular matrix of all animals. They are one of the major components of the basal lamina (one of the layers of the basement membrane). They are important and biologically active in cell differentiation, migration, and adhesion [60] and so play a role in uterine epithelial and extracellular remodeling.

## Conclusion

The main finding was that the apoptosis and proliferation of uterine epithelial cells are tightly controlled cell-specific processes that play a big part in maintaining or preventing the sexual cycle and pregnancy. Our findings suggested that NETA disturbed the normal histological picture of the uterus necessary for implantation, thereby preventing conception in the albino rat.

## Data Availability

The datasets used and/or analysed during the current study are available from the corresponding author on reasonable request.
